# The wide variety of reasons for feeling guilty in adults: findings from a large cross-sectional web-based survey

**DOI:** 10.1186/s40359-022-00908-3

**Published:** 2022-08-12

**Authors:** Tobias Luck, Claudia Luck-Sikorski

**Affiliations:** 1 Faculty of Applied Social Sciences, University of Applied Sciences Erfurt, Altonaer Straße 25, 99085 Erfurt, Germany; 2 Department of Economic and Social Sciences and Institute of Social Medicine, Rehabilitation Sciences and Healthcare Research (ISRV), University of Applied Sciences Nordhausen, Nordhausen, Germany; 3 Research Group COPE – Chronic Diseases and Psychological Health, SRH University of Applied Health Sciences, Gera, Germany; 4 Integrated Research and Treatment Center (IFB) Adiposity Diseases, University Hospital Leipzig, Leipzig, Germany

**Keywords:** Guilt, Feelings of guilt, Feeling guilty, Reasons, Events, Adults, Gender, Age, Adulthood

## Abstract

**Background:**

Experiencing some sort of guilt is a common phenomenon in adulthood. As feeling guilty is usually unpleasant and may even lead to further negative psychological consequences like depression, the aim of this study was to provide comprehensive information on the reasons for such feelings in adults.

**Methods:**

A cross-sectional web-based survey was conducted between May 2019 and April 2020, collecting qualitative information on reasons for feeling guilty in n = 604 adults (mean/SD age = 45.3/16.4 years; n = 346/57.3% women, n = 255/42.2% men and n = 3/0.5% adults without identification with a particular gender). Stated reasons were inductively classified into (super-)/categories, and information on frequency and percentage (total, gender- and age-specific) for each of these (super-)/categories was provided.

**Results:**

Participants altogether stated 1515 reasons for feeling guilty that were classified into 12 supercategories and 49 categories. *“Telling lies/withholding truth/information”* followed by *“Not spending (enough) time with family (members)/Not taking (enough) care of family (members)/not being there for family (members)”* were the most frequent categories of reasons for feeling guilty in the sample. Guilt feelings explicitly referring to *“religious beliefs”* or a *“subjectively perceived more general responsibility’”* (e.g., for society, humankind, problems of the world), by contrast, were of minor importance. Male and female participants as well as participants of different ages showed similarities but also several differences in stated reasons for feeling guilty. Female participants, for example, more often experienced feelings of guilt related to family members, children and to some kind of general responsibility for the wellbeing of others, whereas male participants felt guilty more often because of some kind of misconduct/mistakes being made or because of difficulties in marriage/relationship.

**Conclusions:**

Adults can feel guilty for a wide variety of different reasons. Most reasons seem to be rather concrete (e.g., related to concrete negative self-attributions/flaws or to concrete social situations with concrete individuals). There also seem to be some age- and gender-related differences in reasons for feeling guilty.

## Background

Humans are capable of feeling guilty. In younger children, this capability is considered almost exclusively positive as having an important adaptive function, e.g., for learning prosocial behavior or upholding cooperation [1, 2]. With increasing age, and especially in adults, this picture becomes less clear (for more details, see [3]): On the one hand, feelings of guilt in adulthood can have important adaptive—prosocial—functions as well, e.g., by motivating individuals to repair the damages one has caused and to avoid comparable transgressions in the future [1, 2, 4, 5]. On the other hand, feelings of guilt in adulthood also can be maladaptive. The Operationalized Psychodynamic Diagnostics System (OPD-2), for example, defines guilt conflicts as one of seven important mental conflicts of potential (psychodynamic or even psychopathological) importance [6]. Moreover, individuals can experience very strong, long-lasting or irrational feelings of guilt and, as a consequence, negative health-related outcomes such as depression may evolve [1, 7–9]. When it comes to an observed association between experienced feelings of guilt and depression, it is, however, important to note that there can be a causal relationship in both ways: feelings of guilt may contribute to/cause the development of a depressive disorder, but may also be just a consequence of an already existing depressive disorder (for more details, see [3]). In fact, both the International Classification of Diseases 11th Revision (ICD-11) and the Diagnostic and Statistical Manual of Mental Disorders 5th Edition (DSM-5) consider guilt as a symptom of a depressive disorder [10, 11].

The question of whether there are rather adaptive or maladaptive consequences or both consequences of feelings of guilt in adults certainly requires further attention, as feelings of guilt are a common phenomenon in adulthood. For example, we recently identified a point prevalence of 10.6% (95%-CI = 8.7–12.6) and a lifetime prevalence of 68.5% (95%-CI = 65.6–71.3) for feelings of guilt for the German adult population [3, 12].

An important question that may be linked to the question on potential positive and/or negative consequences of feelings of guilt in adulthood is what adults feel guilty about. On a general basis, individuals can feel guilty, for example, for certain behavior, activity, action or inaction, thoughts, feelings, circumstances, intentions, or goals (e.g., [1, 13, 14]). On a more concrete basis, numerous specific types of guilt have been described. Many of the specific types can be subsumed under the broader term *‘interpersonal guilt’,* emphasizing the relational, social character of guilt (in contrast to—e.g., traditional psychoanalytic—views focusing mainly on an individual’s internal states and intrapsychic processes related to guilt; for an overview, e.g., see [14, 15]). Examples of specific types of interpersonal guilt are *‘survivor guilt’*, *‘separation/disloyalty guilt’*, or *‘omnipotent responsibility guilt’* (e.g., see [13, 14]). Further examples for specified specific types of guilt are *‘guilt in bereavement’*, *‘parental guilt’*, *‘white guilt’*, *‘trauma-related guilt’*, *‘combat-related guilt’*, *‘sex guilt’*, *‘weight-related guilt’* or *guilt in certain disorders such as eating disorders or depression*^1^ (e.g., [16–24]).

Existing empirical studies on feelings of guilt in adults often focus on a specific type of guilt or a selection of certain types of guilt (e.g., types of interpersonal guilt). Moreover, depending on the type(s) of guilt of interest, data are often collected in specific samples of individuals (e.g., bereaved persons, parents, white people, individuals with traumatic experiences, patients with specific disorders, etc.). The aim of this study was to add to such undoubtedly important studies by providing more comprehensive information on reasons for feelings of guilt in adults. Using a cross-sectional web-based survey, we openly asked a large number of adults in Germany about reasons for experienced feelings of guilt. In doing so, we sought to provide an overview of the potential variety and importance of different reasons for feelings of guilt in adults and on potential age- and gender-related differences in such reasons.

## Methods

### Survey

We conducted a cross-sectional web-based survey in Germany from May 2019 through April 2020. The global aim of the survey was to collect comprehensive autobiographical information on guilt experiences to learn more about (i) reasons for feeling guilty in adulthood, (ii) strategies that are used by adults for dealing with the guilt feelings, and (iii) associated factors. Recruitment of participants was supported by Consumerfieldwork GmbH, an independent fieldwork agency providing an actively managed proprietary online panel of registered users for research purposes [25]. Participants recruited by Consumerfieldwork GmbH received financial compensation for participation (1 € for completing the entire survey; 0.1 € for completing only parts the survey). Participants were eligible to participate if they (i) were at least 18 years of age and (ii) had experienced feelings of guilt at least once in their lifetime (inclusion criteria). Eligible participants completed the survey on a secure web-based survey platform (SoSci Survey [26]). At the beginning of the survey, information was provided on the study aims, inclusion criteria, designated use of the collected data, data protection, time required to complete the survey, and contact information for queries. Information was also given that completing the survey was considered consent to participate in the survey and to provide the data for the stated research purposes.

The survey included a standardized questionnaire on sociodemographic characteristics, feelings of guilt, depressive features, self-esteem, satisfaction with life, optimism and pessimism. For this report, we analyzed data on participants’ age, gender, and experienced feelings of guilt. Regarding experienced feelings of guilt, we asked participants to write about ongoing guilt experiences as well as past guilt experiences using the following questions:

- (1a) *‘Do you currently have feelings of guilt?’* (Yes/No)
- (1b) If yes: *‘What is the reason or what are the reasons for your currently experienced feelings of guilt?’* (The following information was additionally provided: *‘By using the following table, please state in note form the reason(s) for your currently experienced feelings of guilt. If you are currently experiencing feelings of guilt for several reasons, please state the reasons separately (reason 1, reason 2, *etc*.).’*; This information was followed by a table with the heading *´Reasons for* *currently* *experienced feelings of guilt`* and 10 lines (‘Reason 1: …’ to ‘Reason 10: …’).)
- (2a) *‘Have you ever had feelings of guilt in the past of your life?’* (Yes/No)
- (2b) If yes: ‘*What has been the reason or what have been the reasons for your in the past experienced feelings of guilt?’* (The following information was additionally provided: *‘By using the following table, please state in note form the reason(s) for your in the past experienced feelings of guilt. If you have experienced feelings of guilt for several reasons in the past, please state the reasons separately (reason 1, reason 2 *etc*.).’*; This information was followed by a table with the heading *´Reasons for* *in the past* *experienced feelings of guilt`* and 10 lines (‘Reason 1: …’ to ‘Reason 10: …’).)

### Analysis

In a first step, data were sifted to exclude data sets of all participants not fulfilling inclusion criterion of having experienced feelings of guilt at least once in the lifetime (answering no to both of the questions: *‘Do you currently have feelings of guilt?’; ‘Have you ever had feelings of guilt in the past of your life?’*) and of all participants who fulfilled the inclusion criterion but stated no reason for feeling guilty.

In a second step, the number of stated reasons for feeling guilty *(in the total sample as well as gender- and age-specific)* was determined. To analyze differences in the number of stated reasons between gender and age groups, the Kruskal–Wallis H test with Dunn-Bonferroni post hoc test for multiple pairwise comparisons was applied. All analyses employed an alpha level for statistical significance of 0.05 (two-tailed).

In the third step, stated reasons for feeling guilty were inductively classified into categories (data-driven; not hypotheses-driven). According to common procedures, classification was done by two researchers (the two authors of this article). At every step of the categorization process, the two researchers first analyzed the data separately, and then afterwards discussed the individual results until agreement was reached (e.g., see [27]). More specifically, first, both researchers independently sifted the stated reasons for feeling guilty, each defining a set of categories for classification. Second, the two sets of categories were compared, and a joint consented set of categories was defined. Third, both researchers independently assigned the stated reasons for feeling guilty to the joint set of categories. Fourth, the results of the independent categorization were compared and discussed until an agreement on all assignments was reached. Fifth, both researchers independently sifted the defined set of categories for reasons of feeling guilty, each defining a set of superordinated supercategories for clustering the categories. Finally, the two sets of supercategories were compared, and a joint consented set of supercategories was defined. Some categories could not be further grouped into supercategories. These categories were also considered supercategories.

In a final step, the frequency and percentage for each of the formed categories and supercategories of reasons for feeling guilty *(in the total sample as well as gender- and age-specific)* were calculated. Given the large number of formed (super)categories of reasons for feeling guilty and the small number of cases in a high number of such categories (especially in certain age- and gender-groups) (see below), we were not able to perform any convincing inferential statistical analyses on observed group differences in the frequency/percentage of the formed (super-)categories of reasons for feeling guilty. Generalizations about the German general adult population therefore have to be made with caution (see also the section on limitations below).

All statistical analyses were performed using IBM SPSS Statistics for Windows, Version 27.0.

## Results

### Sample

Altogether, n = 893 adults participated in the survey. Information on n = 15 participants (1.7%) had to be excluded from the analysis because of missing information on feelings of guilt. Another n = 216 participants (24.2%) did not fulfil the inclusion criterion of having experienced feelings of guilt at least once in the lifetime (answering no to both of the questions: *‘Do you currently have feelings of guilt?’*; *‘Have you ever had feelings of guilt in the past of your life?’;* this high proportion of participants who stated that they have never experienced feelings of guilt in their life is in line with our recently identified lifetime prevalence of 68.5%/95%-CI = 65.6–71.3 for feelings of guilt for the German adult population [12]). The remaining n = 662 participants (74.1%) stated that they either currently experienced feelings of guilt or experienced feelings of guilt in the past or both. Among these n = 662 participants, n = 58 (8.8%) stated no reasons for experienced feelings of guilt. Thus, the results of this report are based on collected data of n = 604 adults: n = 346 women (57.3%), n = 255 men (42.2%) and n = 3 adults (0.5%) without personal identification with a particular gender. The mean age of the sample was 45.3 years (SD = 16.4 years; range = 18–84 years).

### Reasons for feeling guilty

#### Number of reasons for feeling guilty

**Fig. 1:**
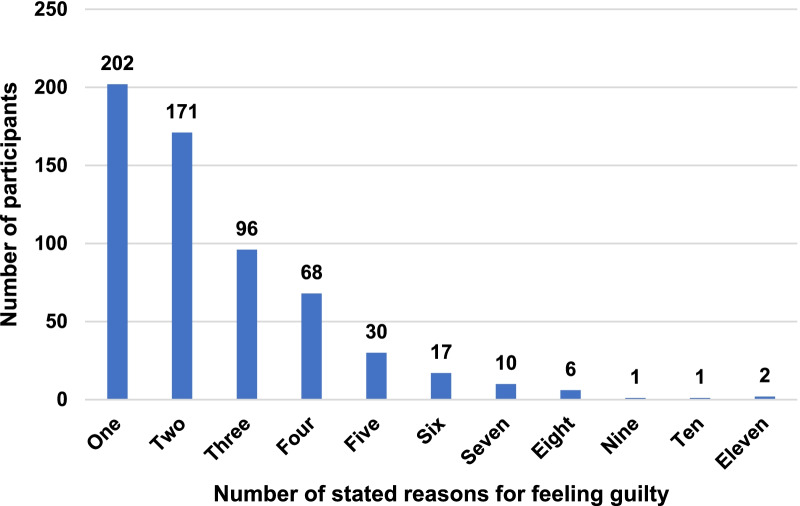
Number of stated reasons for feeling guilty

On average, the n = 604 participants stated 2.5 reasons for experienced feelings of guilt (SD = 1.7; median = 2; range = 1–11; sum = 1515). One-third of the sample (n = 202; 33.4%) stated only one reason, 28.3% (n = 171) stated two reasons, 15.9% (n = 96) stated three reasons, and 22.4% (n = 135) stated four to eleven reasons for feeling guilty (see Fig. 1).

Male participants, on average, stated 2.2 (SD = 1.5) reasons for feeling guilty, female participants stated 2.7 (SD = 1.8) reasons and participants without personal identification with a particular gender stated 5.3 (SD = 3.8) reasons. Only the difference in the number of reasons between male and female participants was statistically significant, keeping, however, in mind that only three adults without personal identification with a particular gender participated in the study (Overall Kruskal–Wallis H test: *χ*^2^ = 11.089, *df* = 2, *p* = 0.004; Dunn-Bonferroni post hoc test for multiple pairwise comparisons: male vs. female participants: *p* = 0.007; male participants versus participants without a particular gender: p = 0.361; female participants versus participants without a particular gender: *p* = 0.788).

To analyze potential differences in the number of reasons stated for feeling guilty by age, we divided the sample into four age groups: an age group of *‘younger adults’* being 18–29 years (*n* = 126; this age group is considered as the so-called *‘emerging adulthood’*; e.g., see [28]), an age group of *‘older adults’* being 60 years or older (n = 151; as there is no universally accepted definition of older age, we followed the United Nations by defining an older person as being someone who is over 60 years of age; e.g., see [29]), and—because of the large age span of 30 years between our classified younger and older age group—two age groups of *‘middle-aged adults’*: adults being 30–44 years (n = 177; *‘younger middle-aged adults’*) and adults being 45–59 years (n = 150; *‘older middle-aged adults’*). Participants aged 18–29 years, on average, stated 2.9 (SD = 1.6) reasons for feeling guilty, participants aged 30–44 years 2.7 (SD = 1.7) reasons, participants aged 45–59 years 2.3 (SD = 1.8) reasons, and participants aged 60 years or older 2.2 (SD = 1.5) reasons. Kruskal–Wallis H test with Dunn-Bonferroni post hoc test for multiple pairwise comparisons showed statistical significance for the higher average numbers of stated reasons for feeling guilty in participants of the younger age groups (18–29, 30–44) compared to participants of the older age groups (45–59, 60 +) (Overall Kruskal–Wallis H test: *χ*^2^ = 25.537, *df* = 3, *p* < 0.001; for Dunn-Bonferroni post hoc test results, see Table 1).

**Table 1:** Dunn-Bonferroni post hoc analysis for multiple pairwise comparisons—number of reasons for feeling guilty by age group (n = 604)

Sample 1–sample 2 comparison	*χ*^2^	Standard error	*p* value^1^
60+ versus 45–59 years	2.591	19.445	1.000
60+ versus 30–44 years	54.009	18.686	0.023
60+ versus 18–29 years	85.426	20.352	< 0.001
45–59 versus 30–44 years	51.418	18.719	0.036
45–59 versus. 18–29 years	82.835	20.383	< 0.001
30–44 versus 18–29 years	31.417	19.661	0.660

^1^With Bonferoni adjustment for multiple comparisons

#### Formed supercategories and categories of reasons for feeling guilty

Altogether, we classified the 1515 reasons for feeling guilty stated by the n = 604 participants into 12 supercategories with 49 more specific categories. Table 2 provides an overview of these supercategories/categories as well as on corresponding examples of actual statements by the study sample. The supercategories and subordinated categories cover feelings of guilt related *to partner/spouse* (category 1), *to child(ren)* (2), to *family members* (3), *to other people (not specifically spouse/partner, children or family members)* (4), *to animals* (5), *to a subjectively perceived responsibility* (6), *to religious beliefs (committing sins; misconduct in the eyes of God)* (7), *to certain circumstances* (8), *to misconduct/mistakes being made* (9), *to decisions (having made bad/ wrong decisions; regretting decisions; being uncertain about decisions)* (10), and *to negative self-attributions/flaws* (11). A final supercategory (12) includes all *general, unspecific, or vaguely formulated reasons for feelings of guilt* (e.g., *“General reasons”*; *“Life in general.”*). Information on the frequency and percentage *(total, gender- and age-specific)* of stated reasons for feeling guilty regarding each of the formed supercategories and categories is provided in Table 3.

**Table 2:** Supercategories/categories of stated reasons for feeling guilty

Supercategory/category	Examples of statements made by the study participants
1 Feelings of guilt related to partner/spouse	
1.1 Divorce/break up	*“Breaking-up with partner.”; “Leaving my wife for a colleague.”*
1.2 Cheating/having affair(s)	*“I cheated on my husband.”; “I kissed a woman other than my partner.”*
1.3 Changing/lacking feelings in relationship/marriage	*“I started a relationship without feelings.”; “I don’t love my husband and just stand everything.”*
1.4 Problems/issues in relationship/marriage *(general; other than 1.1–1.3)*	*“Neglected my husband.”; “Spending too much time for own interests in marriage.”*
2 Feelings of guilt related to child(ren)	
2.1 Termination of contact to child(ren)	*“Having no contact to my daughter.”; “Haven’t seen my daughter for 25 years.”*
2.2 Not spending (enough) time with child(ren)/Not taking (enough) care of child(ren)/not being there for child(ren)	*“Not enough time spent with my child.”; “Going back to work soon after giving birth.”*
2.3 Faults in education/misbehavior towards child(ren)	*“Being too strict with my children.”; “Smoked in the presence of my children.”*
2.4 Certain living conditions of child(ren)	*“My son is a child of divorce.”; “Children have to manage with little money.”*
2.5 Other/unspecific reasons for feelings of guilt towards child(ren)	*“I feel guilty, when I cannot keep my promises to my children.”; “Feelings of guilt towards my children.”*
3 Feelings of guilt related to family (members)	
3.1 Termination of contact to family (members)	*“No contact to family.”; “No contact to sister.”*
3.2 Not spending (enough) time with family (members)/Not taking (enough) care of family (members)/not being there for family (members)	*“Do not take enough care for my widowed mother.”; “Don’t have enough time for my family.”*
3.3 Misbehavior towards/bad thinking of family (members)	*“I treated my parents disrespectfully.”; “Did hurt brother.”*
3.4 Conflict/argument with family (members)	*“Argument with my sister.”; “Conflicts with my parents.”*
3.5 Disappointing/belying expectations of family (members)	*“My parents pay a lot of money for my university study, but I have bad grades.”; “Having disappointed my father.”*
3.6 Certain living conditions of family (members)	*“Mother in nursing home.”; “Had to move far away because of my job – bad for family.”*
3.7 Other/unspecific reasons for feelings of guilt towards family (members)	*“Family matters.”; “Situation in family.”*
4 Feelings of guilt related to other people *(not specifically spouse/partner, children or family members)*	
4.1 Neglecting someone/not taking (enough) care of someone/not being there for someone	*“I neglected a friend.“; “Supported friends not enough.“*
4.2 Misbehavior towards/bad thinking of someone	*“Taking advantage of people.”; “I said hurtful words to someone.”*
4.3 Conflict/argument/communication problem with someone	*“Argument with a person.”; “Argument with friends.”*
4.4 Disappointing/Belying expectations of someone	*“Having disappointed someone.”; “Because of the feeling of not belying the expectations of others.”*
4.5 Not helping someone	*“I promised someone to help moving, but then I didn’t do it.”; “Didn’t help a person, I was close to.”*
4.6 Feelings of guilt related to people at work (*colleagues, employees, superiors, clients, customers *etc*.*)	*“Dispute with coworker.“; “I am not sufficiently available for all of my customers.”*
5 Feelings of guilt related to animals^1^	*“I was cruel to animals in my childhood.“; “The death of my she-dog last year.”*
6 Feelings of guilt related to a subjectively perceived responsibility	
6.1 For life *(situations, events, circumstances)* and death *(circumstances)* of others/for not being able to help/support	*“I couldn’t prevent the suicide of my mother.”; “For the severe heart disease of my child.”*
6.2 For own diseases/own disorders/own traumatic experiences	*“I am sick too often.”; “Didn’t defend myself against being raped.“*
6.3 For surviving	*“I survived cancer. Others are dying of it.“; “My brother died when he was a kid and I live.“*
6.4 For having a better life than other people/Doing not enough against the problems on the world	*“That I live at the expense of the people in the Third World.”; “Doing little for the environment.“*
7 Feelings of guilt related to religious beliefs (*committing sins; misconduct in the eyes of God*)^1^	*“I broke God’s commandments.“; “Sins.”*
8 Feelings of guilt related to certain circumstances	
8.1 Being dependent on someone	*“Living on my parents’ money.”; “Because always someone has to give me ride.“*
8.2 Financial situation/debts/handling of finances	*“I owed a friend money.”; “Credits.”*
8.3 Situation/problems at work	*“I didn`t do my job properly.”; “Didn’t finish my work in time.”*
8.4 Unemployment/incapacity for work/early retirement	*“Lost my job.”; “Because I’m unemployed and don’t find a job.”*
9 Feelings of guilt related to misconduct/mistakes being made	
9.1 Stealing something	*“Stole a toy from my cousin.”; “Stole something from a store, when I was a kid.”*
9.2 Criminal acts/infringement *(not or not specifically stealing)*	*“Criminal behaviour.”; “Fraud.”*
9.3 Telling lies/withholding truth/information	*“Lied to my best friend.”; “Concealment of an event that has taken place and subsequent lies.”*
9.4 Betraying someone/being indiscrete/committing a breach of confidence	*“Gave away a secret.”; “Betrayal.”*
9.5 Self-inflicted accidents/damages	*“Self-inflicted car accident.”; “Car accident – talked too much as co-driver – driver was distracted.”*
9.6 Other/unspecific misconduct/mistakes being made	*“Behaved unethically.”; “Misconduct.”*
10 Feelings of guilt related to decisions *(having made bad/ wrong decisions; regretting decisions; being uncertain about decisions*)^1^	*“I made the wrong decision concerning my profession.“; “Abortion.”*
11 Feelings of guilt related to negative self-attributions/flaws	
11.1 Not achieving something/failure	*“I do not make something of my life (abandoned several study programs, no vocational training….”; “I didn’t finish my dissertation.”*
11.2 Procrastination/waste of time/being lazy/inactive/powerless/ unmotivated	*“Didn’t help enough with the housework.”; “Procrastination concerning important tasks.”*
11.3 Unfavourable health behavior/self-indulgence	*“I do smoke.”; “I don’t take enough care for my body.”*
11.4 Being unable to cope with something	*“Cannot handle the disease of my husband.”; “My son – 17 – has Asperger’s syndrome. Because of him, I am often stretched to my limits. Sometimes, I would like to put him into a facility.”*
11.5 Being unpunctional/unreliable/forgetful	*“Unpunctuality.”; “Forgetting important things/appointments.”*
11.6 Being selfish/egoistic/egocentric	*“Egoism.”; “I am too self-centered.”*
11.7 Being not empathetic	*“Not enough empathy.”; “Lack of empathy.”*
11.8 Being unfair	*“Being not fair.”; “Unfairness.”*
11.9 Being envious/jealous	*“Being joyful, when I have better grades than my fellow students.”; “Sibling jealousy.”*
11.10 Being impulsive/angry	*“Being impulsive against certain people.”; “When I sometimes got hot-tempered and thus couldn’t control myself.”*
11.11 Being anxious	*“Fears of failure.”; “Being anxious.”*
11.12 Being dissatisfied	*“Dissatisfaction.”; “Self-inflicted dissatisfaction.”*
11.13 Other/unspecific negative self-attributions/flaws	*“Having been naïve and trustful.”; “Personality.”*
12 General, unspecific, or vaguely formulated reasons for feelings of guilt^1^	*“General reasons.”; “Life in general.”*

^1^Supercategories that could not be further divided into categories

**Table 3:** Frequency and percentage of stated reasons for feeling guilty per supercategory/category (n = 604)

Supercategory/category	By gender			By age				Total
	Male participants	Female participants	*^1^	18–29 years	30–44 years	45–59 years	60 years or older	
	No. of stated reasons (%)	No. of stated reasons (%)	No. of stated reasons (%)	No. of stated reasons (%)	No. of stated reasons (%)	No. of stated reasons (%)	No. of stated reasons (%)	No. of stated reasons (%)
1 Feelings of guilt related to partner/spouse	**79 (13.8)**	**75 (8.1)**	**1 (6.3)**	**24 (6.6)**	**56 (11.9)**	**33 (9.6)**	**42 (12.6)**	**155 (10.2)**
1.1 Divorce/break up	23 (4.0)	25 (2.7)	0 (0.0)	5 (1.4)	17 (3.6)	9 (2.6)	17 (5.1)	48 (3.2)
1.2 Cheating/having affair(s)	25 (4.4)	23 (2.5)	0 (0.0)	9 (2.5)	20 (4.2)	6 (1.7)	13 (3.9)	48 (3.2)
1.3 Changing/lacking feelings in relationship/marriage	5 (0.9)	6 (0.6)	0 (0.0)	3 (0.8)	3 (0.6)	4 (1.2)	1 (0.3)	11 (0.7)
1.4 Problems/issues in relationship/marriage *(general; other than 1.1–1.3)*	26 (4.5)	21 (2.3)	1 (6.3)	7 (1.9)	16 (3.4)	14 (4.1)	11 (3.3)	48 (3.2)
2 Feelings of guilt related to child(ren)	**27 (4.7)**	**96 (10.4)**	**0 (0.0)**	**4 (1.1)**	**33 (7.0)**	**41 (11.9)**	**45 (13.5)**	**123 (8.1)**
2.1 Termination of contact to child(ren)	3 (0.5)	2 (0.2)	0 (0.0)	0 (0.0)	0 (0.0)	1 (0.3)	4 (1.2)	5 (0.3)
2.2 Not spending (enough) time with child(ren)/Not taking (enough) care of child(ren)/not being there for child(ren)	11 (1.9)	30 (3.2)	0 (0.0)	1 (0.3)	11 (2.3)	15 (4.3)	14 (4.2)	41 (2.7)
2.3 Faults in education/misbehavior towards child(ren)	5 (0.9)	31 (3.3)	0 (0.0)	3 (0.8)	7 (1.5)	12 (3.5)	14 (4.2)	36 (2.4)
2.4 Certain living conditions of child(ren)	6 (1.0)	17 (1.8)	0 (0.0)	0 (0.0)	10 (2.1)	7 (2.0)	6 (1.8)	23 (1.5)
2.5 Other/unspecific reasons for feelings of guilt towards child(ren)	2 (0.3)	16 (1.7)	0 (0)	0 (0.0)	5 (1.1)	6 (1.7)	7 (2.1)	18 (1.2)
3 Feelings of guilt related to family (members)	**62 (10.8)**	**143 (15.4)**	**4 (25.0)**	**31 (8.5)**	**64 (13.6)**	**55 (15.9)**	**59 (17.7)**	**209 (13.8)**
3.1 Termination of contact to family (members)	3 (0.5)	5 (0.5)	0 (0.0)	0 (0.0)	3 (0.6)	2 (0.6)	3 (0.9)	8 (0.5)
3.2 Not spending (enough) time with family (members)/Not taking (enough) care of family (members)/not being there for family (members)	28 (4.9)	71 (7.7)	2 (12.5)	15 (4.1)	29 (6.1)	29 (8.4)	28 (8.4)	101 (6.7)
3.3 Misbehavior towards/bad thinking of family (members)	7 (1.2)	18 (1.9)	0 (0.0)	2 (0.5)	8 (1.7)	5 (1.4)	10 (3.0)	25 (1.7)
3.4 Conflict/argument with family (members)	7 (1.2)	14 (1.5)	0 (0.0)	5 (1.4)	6 (1.3)	6 (1.7)	4 (1.2)	21 (1.4)
3.5 Disappointing/belying expectations of family (members)	7 (1.2)	18 (1.9)	2 (12.5)	7 (1.9)	9 (1.9)	7 (2.0)	4 (1.2)	27 (1.8)
3.6 Certain living conditions of family (members)	4 (0.7)	4 (0.4)	0 (0.0)	1 (0.3)	1 (0.2)	3 (0.9)	3 (0.9)	8 (0.5)
3.7 Other/unspecific reasons for feelings of guilt towards family (members)	6 (1.0)	13 (1.4)	0 (0.0)	1 (0.3)	8 (1.7)	3 (0.9)	7 (2.1)	19 (1.3)
4 Feelings of guilt related to other people *(not specifically spouse/partner, children or family members)*	**79 (13.8)**	**148 (16.0)**	**3 (18.8)**	**88 (24.1)**	**68 (14.4)**	**50 (14.5)**	**24 (7.2)**	**230 (15.2)**
4.1 Neglecting someone/not taking (enough) care of someone/not being there for someone	13 (2.3)	44 (4.8)	0 (0.0)	18 (4.9)	16 (3.4)	15 (4.3)	8 (2.4)	57 (3.8)
4.2 Misbehavior towards/bad thinking of someone	43 (7.5)	50 (5.4)	3 (18.8)	44 (12.1)	24 (5.1)	21 (6.1)	7 (2.1)	96 (6.3)
4.3 Conflict/argument/communication problem with someone	8 (1.4)	12 (1.3)	0 (0.0)	7 (1.9)	8 (1.7)	5 (1.4)	0 (0.0)	20 (1.3)
4.4 Disappointing/Belying expectations of someone	4 (0.7)	13 (1.4)	0 (0.0)	7 (1.9)	8 (1.7)	0 (0.0)	2 (0.6)	17 (1.1)
4.5 Not helping someone	5 (0.9)	15 (1.6)	0 (0.0)	9 (2.5)	4 (0.8)	4 (1.2)	3 (0.9)	20 (1.3)
4.6 Feelings of guilt related to people at work *(colleagues, employees, superiors, clients, customers *etc*.)*	6 (1.0)	14 (1.5)	0 (0.0)	3 (0.8)	8 (1.7)	5 (1.4)	4 (1.2)	20 (1.3)
5 Feelings of guilt related to animals^2^	**5 (0.9)**	**26 (2.8)**	**0 (0.0)**	**7 (1.9)**	**7 (1.5)**	**8 (2.3)**	**9 (2.7)**	**31 (2.0)**
6 Feelings of guilt related to a subjectively perceived responsibility	**37 (6.5)**	**92 (9.9)**	**2 (12.5)**	**32 (8.8)**	**23 (4.9)**	**41 (11.9)**	**35 (10.5)**	**131 (8.6)**
6.1 For life *(situations, events, circumstances)* and death *(circumstances)* of others/for not being able to help/support	18 (3.1)	55 (5.9)	0 (0.0)	23 (6.3)	10 (2.1)	19 (5.5)	21 (6.3)	73 (4.8)
6.2 For own diseases/own disorders/own traumatic experiences	4 (0.7)	20 (2.2)	1 (6.3)	4 (1.1)	9 (1.9)	7 (2.0)	5 (1.5)	25 (1.7)
6.3 For surviving	0 (0.0)	2 (0.2)	0 (0.0)	0 (0.0)	0 (0.0)	1 (0.3)	1 (0.3)	2 (0.1)
6.4 For having a better life than other people/Doing not enough against the problems on the world	15 (2.6)	15 (1.6)	1 (6.3)	5 (1.4)	4 (0.8)	14 (4.1)	8 (2.4)	31 (2.0)
7 Feelings of guilt related to religious beliefs *(committing sins; misconduct in the eyes of God)*^2^	**2 (0.3)**	**4 (0.4)**	**0 (0.0)**	**3 (0.8)**	**1 (0.2)**	**1 (0.3)**	**1 (0.3)**	**6 (0.4)**
8 Feelings of guilt related to certain circumstances	**38 (6.6)**	**36 (3.9)**	**2 (12.5)**	**19 (5.2)**	**24 (5.1)**	**23 (6.7)**	**10 (3.0)**	**76 (5.0)**
8.1 Being dependent on someone	0 (0.0)	6 (0.6)	2 (12.5)	6 (1.6)	1 (0.2)	1 (0.3)	0 (0.0)	8 (0.5)
8.2 Financial situation/debts/handling of finances	18 (3.1)	8 (0.9)	0 (0.0)	2 (0.5)	7 (1.5)	12 (3.5)	5 (1.5)	26 (1.7)
8.3 Situation/problems at work	16 (2.8)	16 (1.7)	0 (0.0)	10 (2.7)	14 (3.0)	6 (1.7)	2 (0.6)	32 (2.1)
8.4 Unemployment/incapacity for work/early retirement	4 (0.7)	6 (0.6)	0 (0.0)	1 (0.3)	2 (0.4)	4 (1.2)	3 (0.9)	10 (0.7)
9 Feelings of guilt related to misconduct/mistakes being made	**111 (19.4)**	**107 (11.6)**	**1 (6.3)**	**75 (20.5)**	**80 (16.9)**	**35 (10.1)**	**29 (8.7)**	**219 (14.5)**
9.1 Stealing something	7 (1.2)	7 (0.8)	0 (0.0)	4 (1.1)	4 (0.8)	1 (0.3)	5 (1.5)	14 (0.9)
9.2 Criminal acts/infringement *(not or not specifically stealing)*	9 (1.6)	3 (0.3)	0 (0.0)	2 (0.5)	4 (0.8)	1 (0.3)	5 (1.5)	12 (0.8)
9.3 Telling lies/withholding truth/information	48 (8.4)	71 (7.7)	1 (6.3)	46 (12.6)	44 (9.3)	20 (5.8)	10 (3.0)	120 (7.9)
9.4 Betraying someone/being indiscrete/committing a breach of confidence	8 (1.4)	2 (0.2)	0 (0.0)	5 (1.4)	3 (0.6)	0 (0.0)	2 (0.6)	10 (0.7)
9.5 Self-inflicted accidents/damages	16 (2.8)	8 (0.9)	0 (0.0)	3 (0.8)	11 (2.3)	6 (1.7)	4 (1.2)	24 (1.6)
9.6 Other/unspecific misconduct/mistakes being made	23 (4.0)	16 (1.7)	0 (0.0)	15 (4.1)	14 (3.0)	7 (2.0)	3 (0.9)	39 (2.6)
10 Feelings of guilt related to decisions *(having made bad/ wrong decisions; regretting decisions; being uncertain about decisions)*^2^	**12 (2.1)**	**22 (2.4)**	**0 (0.0)**	**5 (1.4)**	**13 (2.8)**	**7 (2.0)**	**9 (2.7)**	**34 (2.2)**
11 Feelings of guilt related to negative self-attributions/flaws	**101 (17.6)**	**146 (15.8)**	**3 (18.8)**	**67 (18.4)**	**82 (17.4)**	**41 (11.9)**	**60 (18.0)**	**250 (16.5)**
11.1 Not achieving something/failure	19 (3.3)	36 (3.9)	2 (12.5)	13 (3.6)	18 (3.8)	10 (2.9)	16 (4.8)	57 (3.8)
11.2 Procrastination/waste of time/being lazy/inactive/powerless/ unmotivated	13 (2.3)	26 (2.8)	1 (6.3)	13 (3.6)	13 (2.8)	4 (1.2)	10 (3.0)	40 (2.6)
11.3 Unfavourable health behavior/self-indulgence	21 (3.7)	19 (2.1)	0 (0.0)	5 (1.4)	12 (2.5)	10 (2.9)	13 (3.9)	40 (2.6)
11.4 Being unable to cope with something	2 (0.3)	3 (0.3)	0 (0.0)	1 (0.3)	1 (0.2)	2 (0.6)	1 (0.3)	5 (0.3)
11.5 Being unpunctional/unreliable/forgetful	7 (1.2)	11 (1.2)	0 (0.0)	7 (1.9)	7 (1.5)	2 (0.6)	2 (0.6)	18 (1.2)
11.6 Being selfish/egoistic/egocentric	3 (0.5)	5 (0.5)	0 (0.0)	1 (0.3)	6 (1.3)	0 (0.0)	1 (0.3)	8 (0.5)
11.7 Being not empathetic	1 (0.2)	4 (0.4)	0 (0.0)	0 (0.0)	2 (0.4)	1 (0.3)	2 (0.6)	5 (0.3)
11.8 Being unfair	3 (0.5)	1 (0.1)	0 (0.0)	0 (0.0)	3 (0.6)	0 (0.0)	1 (0.3)	4 (0.3)
11.9 Being envious/jealous	0 (0.0)	3 (0.3)	0 (0.0)	3 (0.8)	0 (0.0)	0 (0.0)	0 (0.0)	3 (0.2)
11.10 Being impulsive/angry	3 (0.5)	6 (0.6)	0 (0.0)	2 (0.5)	2 (0.4)	3 (0.9)	2 (0.6)	9 (0.6)
11.11 Being anxious	4 (0.7)	3 (0.3)	0 (0.0)	3 (0.8)	1 (0.2)	2 (0.6)	1 (0.3)	7 (0.5)
11.12 Being dissatisfied	0 (0.0)	3 (0.3)	0 (0.0)	3 (0.8)	0 (0.0)	0 (0.0)	0 (0.0)	3 (0.2)
11.13 Other/unspecific negative self-attributions/flaws	25 (4.4)	26 (2.8)	0 (0.0)	16 (4.4)	17 (3.6)	7 (2.0)	11 (3.3)	51 (3.4)
12 General, unspecific, or vaguely formulated reasons for feelings of guilt^2^	**20 (3.5)**	**31 (3.3)**	**0 (0.0)**	**10 (2.7)**	**21 (4.4)**	**10 (2.9)**	**10 (3.0)**	**51 (3.4)**
Total number of stated reasons	573 (100)	926 (100)	16 (100)	365 (100)	472 (100)	345 (100)	333 (100)	1515 (100)

Bold text refers to the supercategories of reasons for feeling guilty

*^1^Participants without personal identification with a particular gender

^2^Supercategories that could not be further divided into categories

#### Reasons for feeling guilty in the total sample

The supercategory covering the highest number of stated reasons in the total sample was *“Feelings of guilt related to negative self-attributions/flaws”* (supercategory 11; 250 stated reasons; 16.5% of all stated reasons), followed by *“Feelings of guilt related to other people (not specifically spouse/partner, children or family members)”* (4; 230 reasons; 15.2%) and *“Feelings of guilt related to misconduct/mistakes being made”* (9; 219 reasons; 14.5%).

**Fig. 2:**
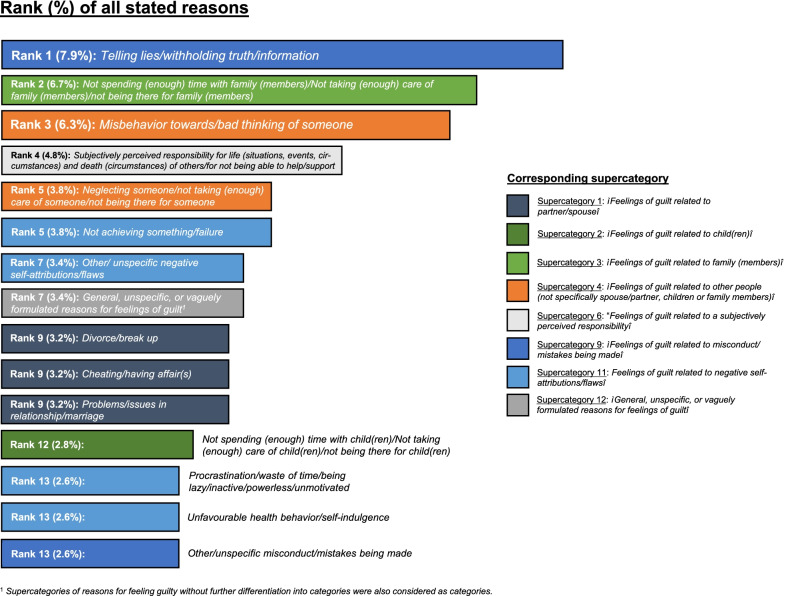
Categories of the most frequently stated reasons for feeling guilty in the total sample

Regarding the more specific categories of reasons for feeling guilty, the highest number of stated reasons were assigned to:

1. *“Telling lies/withholding truth/information”* (category 9.3; 120 stated reasons; 7.9% of all stated reasons),
2. *“Not spending (enough) time with family (members)/Not taking (enough) care of family (members)/not being there for family (members)”* (3.2; 101 reasons; 6.7%), and
3. *“Misbehavior towards/bad thinking of someone”* (4.2; 96 reasons; 6.3%) (*see* Table 3 and Fig. 2*additionally providing a graphical overview of the first 15 categories of the most frequently stated reasons for feeling guilty*)*.*

#### Gender and reasons for feeling guilty

The three participants without personal identification with a particular gender overall stated 16 reasons for feeling guilty. One-fourth of these reasons were assigned to supercategory 3 *“Feelings of guilt related to family (members)”* (4 stated reasons; 25.0% of all reasons stated by the three participants), 3 reasons (18.8%) to supercategory 4 *“Feelings of guilt related to other people (not specifically spouse/partner, children or family members)”* and 3 reasons (18.8%) to supercategory 11 *“Feelings of guilt related to negative self-attributions/flaws”*. The category covering the highest number of reasons stated by the participants without personal identification with a particular gender was 4.2 *“Misbehavior towards/bad thinking of someone”*. (3 reasons; 18.8%) (see Table 3).

The n = 255 male participants stated 573 reasons for feeling guilty, and the n = 346 female participants stated 926 reasons for feeling guilty. Supercategories covering the highest number of reasons stated by the male participants were *“Feelings of guilt related to misconduct/mistakes being made”* (supercategory 9; 111 stated reasons; 19.4% of all reasons stated by the male participants), *“Feelings of guilt related to negative self-attributions/flaws”* (11; 101 reasons; 17.6%), *“Feelings of guilt related to partner/spouse”* (1) and *“Feelings of guilt related to other people (not specifically spouse/partner, children or family members)”* (4; each 79 reasons; 13.8%). Supercategories covering the highest number of reasons stated by the female participants were *“Feelings of guilt related to other people (not specifically spouse/partner, children or family members)”* (4; 148 reasons; 16.0%), *“Feelings of guilt related to negative self-attributions/flaws”* (11; 146 reasons; 15.8%) and *“Feelings of guilt related to family (members)”* (3; 143 reasons; 15.4%) (see Table 3).

**Fig. 3:**
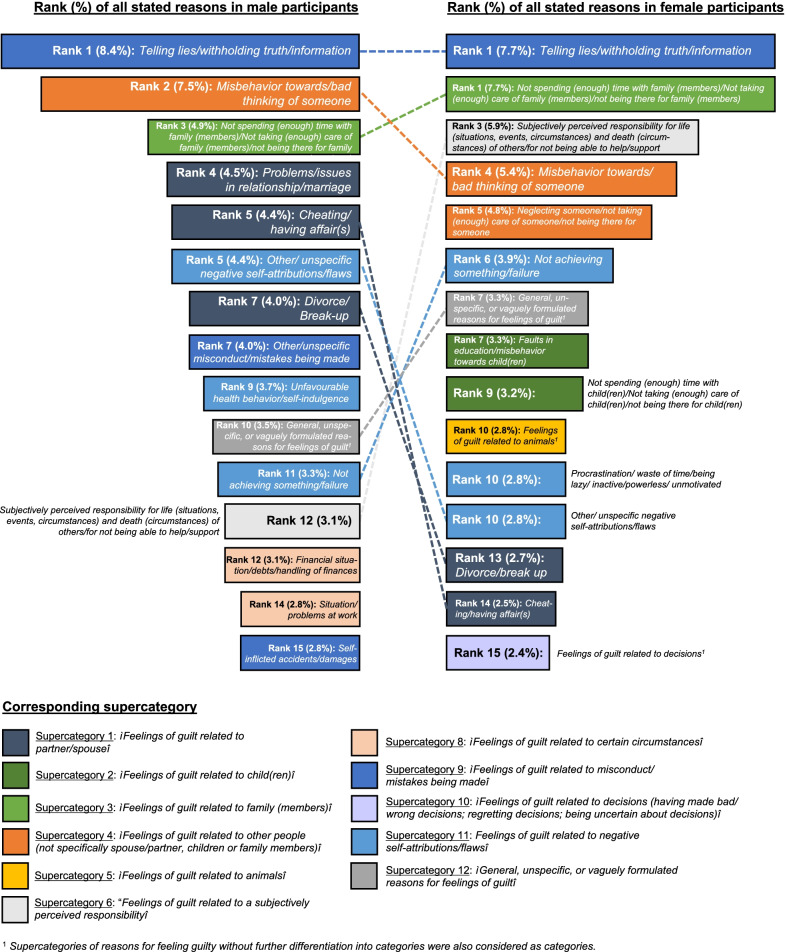
Categories of the most frequently stated reasons for feeling guilty in male and female participants

Regarding the more specific categories of reasons for feeling guilty, the highest number of reasons stated by the male participants were assigned to *“Telling lies/withholding truth/information”* (category 9.3; 48 stated reasons; 8.4% of all reasons stated by the male participants), followed by *“Misbehavior towards/bad thinking of someone”* (4.2; 43 reasons, 7.5%) and *“Not spending (enough) time with family (members)/Not taking (enough) care of family (members)/not being there for family”* (3.2; 28 reasons; 4.9%). These three categories were also very frequent in the female participants (*“Telling lies/withholding truth/information”* and *“Not spending (enough) time with family (members)/Not taking (enough) care of family (members)/not being there for family”* on tied first rank with 71 stated reasons and 7.7% of all reasons stated by the female participants each; *“Misbehavior towards/bad thinking of someone”* on fourth rank with 50 reasons/5.4%) (*see* Table 3 and Fig. 3*additionally providing a graphical overview on similarities and differences between male and female participants in the first 15 categories of stated reasons for feeling guilty*).

Female participants, however, more frequently stated reasons for feeling guilty assigned to categories like 6.1 “*Subjectively perceived responsibility for life (situations, events, circumstances) and death (circumstances) of others/for not being able to help/support”* (rank 3), 4.2 *“Neglecting someone/not taking (enough) care of someone/not being there for someone*” (rank 5), 2.3 *“Faults in education/misbehavior towards child(ren)”* (rank 7) or 2.2 *“Not spending (enough) time with child(ren)/Not taking (enough) care of child(ren)/not being there for child(ren)”* (rank 9) than male participants. Male participants, by contrast, more frequently stated reasons for feeling guilty related to partner/spouse (categories 1.3 *“Problems/issues in relationship/marriage”*, 1.2*”Cheating/having affair(s)”* and 1.1 *“Divorce/Break-up”* on ranks 4, 5 and 7) than female participants.

#### Age and reasons for feeling guilty

Participants in the 18–29 year age group altogether stated 365 reasons for feeling guilty. Approximately one-fourth (n = 88; 24.1%) of these reasons were assigned to supercategory 4 *“Feelings of guilt related to other people (not specifically spouse/partner, children or family members)”*, followed by supercategories 9 *“Feelings of guilt related to misconduct/mistakes being made”* (75 reasons; 20.5%) and *11 “Feelings of guilt related to negative self-attributions/flaws”* (67 reasons; 18.4%). These three supercategories were also the top 3 supercategories in participants in the 30–44 year age group, with supercategory 11 ranking first (82 of 472 reasons for feeling guilty stated by participants in this age group; 17.4%), supercategory 9 ranking second (80 reasons; 16.9%), and supercategory 4 ranking third (68 reasons; 14.4%).

Regarding participants in the 45–59 age group, reasons for feeling guilty were most frequently assigned to supercategories 3 *“Feelings of guilt related to family (members)”* (55 of 345 reasons stated by this age group; 15.9%), 4 *“Feelings of guilt related to other people (not specifically spouse/partner, children or family members)”* (50 reasons; 14.5%) and 2 *“Feelings of guilt related to child(ren)”*, 6 *“Feelings of guilt related to a subjectively perceived responsibility”* and 11 *“Feelings of guilt related to negative self-attributions/flaws”* on tied third rank (each 41 reasons; 11.9%). Three of these five supercategories (2, 3, 11) were also the top 3 supercategories in participants aged 60 years or older, with supercategory 11 again ranked first (60 of 333 reasons for feeling guilty stated by this age group; 18.0%), followed by supercategory 3 ranked second (59 reasons; 17.7%) and supercategory 2 ranked third (45 reasons; 13.5%) (*see* Table 3).

**Fig. 4:**
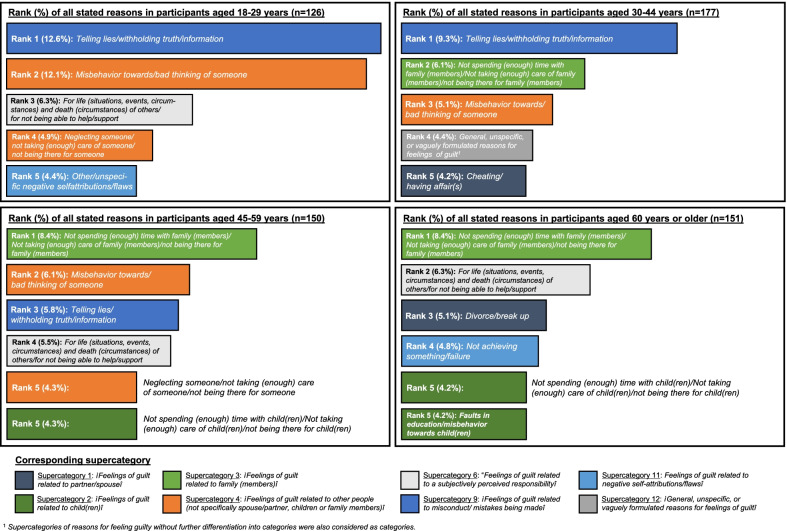
Categories of the most frequently stated reasons for feeling guilty by age group

Regarding the more specific categories of reasons for feeling guilty, category 9.3 *“Telling lies/withholding truth/information”* covered the highest number of stated reasons in participants of the two younger age groups 18–29 years and 30–44 years (46 reasons/12.6% and 44/9.3%) and category 3.2 *“Not spending (enough) time with family (members)/Not taking (enough) care of family (members)/not being there for family (members)”* the highest number of reasons in participants of the two older age groups 45–59 years and 60 years or older (29 reasons/8.4% and 28/8.4%) (*see* Table 3 and Fig. 4*additionally providing a graphical overview on the first five categories of the most frequently stated reasons for feeling guilty in participants of the four different age groups*). *The latter category* was also found to be one of the most important categories (rank 2) in participants in the 30–44 age group but did not reach the top 5 categories in participants in the youngest age group (18–29 years). *“Telling lies/withholding truth/information”*, by contrast, was also found to be important in participants aged 45–59 years (rank 3 in addition to rank 1 in participants aged 18–29 and 30–44 years) but not in participants aged 60 years or older. Comparable age differences can also be seen for category 4.2 *“Misbehavior towards/bad thinking of someone”*. As shown in Table 3 and Fig. 4, this category reached rank 2, 3 and 2 in participants of age groups 18–29, 30–44 and 45–59 years, but it was of less relevance in participants of age group 60 years or older. Participants of the oldest age group of the sample, however, stated reasons for feeling guilty that were, for example, more frequently assigned to categories such as *“Divorce/break up”* (rank 3) or *“Not achieving something/failure”* (rank 4) than in participants of the three younger age groups.

## Discussion

In this report, we sought to provide comprehensive empirical information on reasons for feelings of guilt in adults. Analyzing data from a large sample of n = 604 adults (18–84 years) participating in a cross-sectional web-based survey, we particularly sought to provide an overview of the potential variety and importance of different reasons for feelings of guilt in adults and on potential age- and gender-related differences in such reasons. Several aspects of our identified findings may deserve a closer look:

### Variety and importance of reasons for feeling guilty

First and foremost, as the n = 604 participants stated 1515 reasons for feeling guilty that were classified into 12 supercategories and even 49 categories, our study identified a wide variety of different reasons for feelings of guilt in adults.

As stated in the introduction, there are many potential general sources for feelings of guilt, such as certain behavior, activity, action or inaction, thoughts, feelings, circumstances, intentions, or goals (e.g., [1, 13, 14]). Our findings also reflect these various general sources. By looking more deeply in our findings, ‘*(mis-)behavior’* may be an important general source for feelings of guilt in adulthood, reflecting categories *such as “telling lies/withholding truth/information”* (rank 1 of the categories with the most frequently stated reasons for feeling guilty), *“misbehavior towards/bad thinking of someone”* (rank 3; this category, of course, can be additionally linked to the general source ‘thoughts’) or *“cheating/having affair(s)”* (rank 9). Other highly ranked categories such as *“Not spending (enough) time with family (members)/Not taking (enough) care of family (members)/not being there for family (members)”* (rank 2) *or “Neglecting someone/not taking (enough) care of someone/not being there for someone”* (tied rank 5) or *“Not spending (enough) time with child(ren)/Not taking (enough) care of child(ren)/not being there for child(ren)”* (rank 12) may either also be considered as some kind of *‘(mis-)behavior’* or as *‘inactivity’*. *‘Inactivity’* may be additionally linked, for example, to a category such as *“Procrastination/waste of time/being* lazy/inactive/powerless/unmotivated” (rank 13).

As stated in the introduction as well, numerous specific types of guilt have been described in the literature, of which many can be subsumed under a broader term called *‘interpersonal guilt’*. The term *‘interpersonal guilt’*—emphasizing the relational, social character of guilt (for an overview, e.g., see [14, 15])—may also apply to the majority of identified reasons for feelings of guilt in our study considering, for example, the entire supercategories (and subordinated categories) 1–4 *(“Feelings of guilt related to partner/spouse, child(ren), family (members) and other people”)*. Moreover, some of the described specific types of *‘interpersonal guilt’* may be found in our data:

*‘Omnipotent responsibility guilt’*—a type of guilt that “[…] involves an exaggerated sense of responsibility and concern for the happiness and well-being of others.” (O’Connor et al. [14], p. 76), for example, may be linked to the category *“Subjectively perceived responsibility for life (situations, events, circumstances) and death (circumstances) of others/for not being able to help/support”* (rank 4 of the categories with the most frequently stated reasons for feeling guilty). *‘Survivor guilt’* in its broader sense as a belief that an attainment of good things is not fair to other people who have not attained such good things or is at the expense of those other people [14, 30] may be linked to categories *“Feelings of guilt related to a subjectively perceived responsibility for surviving”* or *“Feelings of guilt related to a subjectively perceived responsibility for having a better life than other people/Doing not enough against the problems on the world”*. *‘Separation/disloyalty guilt’* as a “[…] belief that one is harming one’s parents or other loved ones by separating from them or by differing from them and thereby being disloyal” (O’Connor et al. [14], p. 76; [30]) may be linked at least to some of the stated reasons in categories such as *“Disappointing/belying expectations of family (members)”* or *“Disappointing/Belying expectations of someone”*.

Among the numerous further specific types of guilt that have been described in the literature, *‘parental guilt’* additionally may have to be mentioned here. As *‘parental guilt’* may be defined as a feeling of doing/having done something wrong and/or not enough in parenting in relation to own standards or standards of others, the entire supercategory 2 of identified reasons for feeling guilty in our study (*“Feelings of guilt related to child(ren)”*) may be regarded as some kind of *‘parental guilt’*. Worth mentioning may also be some other specific types of guilt that at least can be linked to some of the stated reasons for feeling guilty in some of the differentiated categories in our study. ‘*Guilt in certain disorders’* and *‘trauma-related guilt’*, for instance, can be linked to stated reasons in category *“Feelings of guilt related to a subjectively perceived responsibility for own diseases/own disorders/own traumatic experiences”*, *‘weight-related* guilt’ to stated reasons in category *“Unfavourable health behaviour/self-indulgence”*, and *‘guilt in bereavement’* to stated reasons in category *“Feelings of guilt related to a subjectively perceived responsibility for life (situations, events, circumstances) and death (circumstances) of others/for not being able to help/support”*. Other specific types of guilt may certainly be important in specific groups of adult individuals/patients and/or in specific situations/constellations but did not play a role in the answers of our more general sample of adults (e.g., *‘combat-related guilt’; see also below in the limitations section*).

Two further findings of our study regarding the variety and importance of reasons for feeling guilty might be noteworthy. First, only a very small number (n = 6; 0.4%) of all stated reasons for feeling guilty explicitly referred to religious beliefs such as committing sins or misconduct in the eyes of God. Surely, many of the other stated reasons may also reflect some internalized religious commandments/norms/values (e.g., cheating/having affairs, stealing something, telling lies/withholding truth/information). The lack of explicit references to religious beliefs, however, may still be quite surprising, as concepts such as *‘guilt’* or *‘sin(s)’* are an integral part of many religious belief systems and as many adults in Germany are still members of a religious community (e.g., 22.6 million members of the Catholic Church, 20.7 million members of the Evangelical Church and 95,000 members of the Jewish community in 2019, and 4.4–4.7 million Muslims in 2015 [31, 32].

The other also noteworthy finding may be the fact that the vast majority of stated reasons for feeling guilty are related to *concrete* negative self-attributions/flaws or to *concrete* social situations, experiences, incidents, (in-)actions, (mis-)behaviors with *concrete* individuals (partner/spouse, child(ren) etc.). Feelings of guilt on a more *universal, global level* (e.g., feelings of guilt related to society or to humankind in general), by contrast, are rather rare considering the small number of stated reasons (n = 31; 2.0% of all stated reasons) in the category “*Feelings of guilt related to a subjectively perceived responsibility for having a better life than other people/Doing not enough against the problems on the world”.* Following the idea of experiencing guilt as an important adaptive prosocial mechanism, the mechanism may be rather limited to the more direct, closer, more experienceable social environment and may be less effective on a higher, more abstract social level. We may, for example, feel less guilty for a certain negative lifestyle (e.g., consumption or environmental behavior) if this lifestyle mainly affects people in other areas/countries/parts of the world but not or not obviously people in our direct social environment.

### Gender and reasons for feeling guilty

As, unfortunately, only three adults without personal identification with a particular gender participated in our study, conclusions on potential differences and similarities in stated reasons for feeling guilty between these three participants and male/female participants would be way too speculative. Thus, we only focus here in the discussion section on the results of the male and female participants.

Regarding these two gender groups, similarities but also several differences in stated reasons for feeling guilty have been found. Both male and female participants, for example, often tended to have feelings of guilt because of *“telling lies/withholding truth/information”*. *“Feelings of guilt related to misconduct/mistakes being made”* in general (the supercategory of stated reasons including the subordinated category *“Telling lies/withholding truth/information”*), however, were more frequent in male than in female participants. It might be speculated that the reasons classified in this supercategory describing wrongdoings, deviant and to some extent even delinquent behavior (*“Stealing something”*, *“Criminal acts/infringement”,* etc.) simply reflect usually identified empirical gender differences in the frequency of such behaviors. To provide one example for a related statistic, in 2020, 75.2% of all suspects in criminal acts in Germany were men and 24.8% women [33].

Returning to some similarities, both male and female participants frequently experienced feelings of guilt because of *“Not spending (enough) time with family (members)/Not taking (enough) care of family (members)/not being there for family”*. The more general supercategory *“Feelings of guilt related to family (members)”,* including these reasons for feeling guilty, however, was more frequent in female participants. Female participants also more often experienced feelings of guilt related to children (e.g., *“Faults in education/misbehavior towards child(ren”; “Not spending (enough) time with child(ren)/Not taking (enough) care of child(ren)/not being there for child(ren)”*) and to some kind of general responsibility for the wellbeing of others (“*Subjectively perceived responsibility for life (situations, events, circumstances) and death (circumstances) of others/for not being able to help/support”*; *“Neglecting someone/not taking (enough) care of someone/not being there for someone*”), whereas male participants felt guilty more often related to the partner/spouse *(see the corresponding supercategory “Feelings of guilt related to partner/spouse” including categories like “Problems/issues in relationship/marriage”*,*”Cheating/having affair(s)”* and *“Divorce/ Break-up”* in Table 3 and Fig. 3).

It might be speculated that the higher frequency of reasons for feeling guilty of female participants regarding family members, children, and relevant others may also be to some extent a consequence of corresponding empirical gender differences. Indeed, women in Germany, on average, spend 52.4% more time per day on unpaid care work (e.g., for households and gardening or for the care of children and adults) than men [34]. It might be assumed that the more things you have to care for the more ‘opportunities’ for feeling guilty will appear. Moreover, it is also thinkable that the gender differences in these particular reasons for feeling guilty may reflect some (internalized) traditional expectations/norms of society (e.g., *“You are only a good mother, if you…”*; *“You have to take care of your wife…”*).

Even though it is important to look for potential differences in outcomes with regard to an important sociodemographic characteristic such as gender and to find reasonable explanations for identified gender differences, such differences should also not be overinterpreted or overemphasized. Regarding our findings on the outcome *‘reasons for feeling guilty’*, for example, it is important to note that—despite the fact that we have found differences between male and female participants—these are just differences in the frequency; all classified supercategories (12 out of 12) and almost all classified categories (45 out of 49) of stated reasons were found in both male and female participants. By borrowing heavily from the title of the very important study of Carothers and Reis on the latent structure of gender: Men are not from Mars and women are not from Venus—“Men and women are from Earth” ([35]; p. 385).

### Age and reasons for feeling guilty

The risk of overinterpreting or overemphasizing, of course, also applies to possible age differences in reasons for feeling guilty. Notwithstanding, we carefully want to address some of the identified differences:

*“Telling lies/withholding truth/information”* and *“misbehavior towards/bad thinking of someone”*, for example, were found to be very frequent categories of stated reasons for feeling guilty in participants of the three younger age groups 18–29, 33–44 and 45–59 years but rather rare categories in participants of the oldest age group 60 years or older. Several reasons are thinkable for the lower importance of these reasons for feeling guilty in older age. First, it is possible that the older generation indeed lied less frequently, thought less frequently bad of others or misbehaved less frequently throughout the lifespan than the younger generations and thus also experienced fewer feelings of guilt. Such better behaviors, for example, might be attributed to an assumed more conservative, more value-based education in the times the older participants were young.

A second, probably more convincing, reason may be a significant change in social relationships and the social environment with older age (for an overview, see [36]): (i) Social roles generating stress are reduced. The work environment as an important, hardly avoidable source of interpersonal problems does not matter anymore in regard to retirement. (ii) With increasing age, adults are optimizing their social relationships. Relationships that are more rewarding are actively sought, and relationships that are less rewarding are actively disbanded. (iii) […] “[O]lder adults appraise their social relationships more positively, even in the face of negative social exchanges” ([36]; p. 12). (iv) Older adults are usually more socially experienced and thus make better judgments regarding potential social partners (e.g., avoiding social partners with a higher risk of confrontations). (v) Older adults usually avoid conflicts more often/more effectively than younger adults by using so-called *‘disengagement strategies’*, such as ignoring a negative situation or avoiding the topic of a conflict [37]. (vi) Social partners and society as a whole may treat older adults kindlier. (vii) And finally, especially in older age, adults often have fewer social contacts or are even at risk of being socially isolated (e.g., because spouse/partner/family members/friends have passed away or because children are living somewhere else). As a result, older adults might have simply less opportunities to misbehave than younger ones. These and other changes and mechanisms may lead to fewer ‘reasons’ for *“telling lies/withholding truth/information”* and *“misbehavior towards/bad thinking of someone”* and thus to less associated feelings of guilt in older age.

However, what about such potential feelings in a time when the older participants were younger and social relationships and the social environment haven’t/hasn’t changed yet? Should the older participants not remember their feelings of guilt experienced at a younger age? One answer may be that memory is not static. Humans forget. Situations in life can be re-evaluated, and associated emotions can change over time. In fact, a so-called *‘fading affect bias’* has been demonstrated in several studies, indicating that the intensity of an emotion being associated with a negative autobiographical memory fades faster than the emotion being associated with a positive one (e.g., [38, 39]). This psychological effect, though important, for example, for promoting a positive self-concept may additionally explain the lower frequency of feelings of guilt related to rather negative behaviors such as “*Telling lies/withholding truth/information”* and *“Misbehavior towards/bad thinking of someone”* and the generally identified lower average numbers of stated reasons for feeling guilty in participants of the older (45–59, 60+) compared to participants of the younger age groups (18–29, 30–44).

Explanations for other identified age differences in reasons for feeling guilty may be less comprehensive and complex. The lower frequency of stated reasons for feeling guilty related to *“Not spending (enough) time with family (members)/Not taking (enough) care of family (members)/not being there for family (members)”* in participants of age group 18–29 years than in participants of the three older age groups, for example, may be explained by the fact that other things in this age usually are more important like finishing school, doing an apprenticeship, studying at a college/university or finding a partner. According to the famous psychoanalytic theory of Erik H. Erikson describing eight different stages of psychosocial development throughout the lifespan, the major ‘task’ in young adulthood is to form intimate, loving relationships with other people (Stage 6: ‘*Intimacy vs. Isolation’*), whereas being there for others becomes an important task later in life (Stage 7: *‘Generativity vs. Stagnation’*; middle adulthood) [40].

Findings of a higher frequency of stated reasons for feeling guilty with regard to *“Divorce/break up” and “Not achieving something/failure”* in participants of the oldest age group 60+ years, by contrast, fits perfectly to the last—eighth—stage of Erikson’s theory. According to Erikson, at this stage (*‘Integrity vs. Despair’*; late adulthood), the major task of an individual is to be able to look back on his or her own life with a sense of accomplishment and fulfilment (integrity). By looking back, however, some people may experience negative feelings such as disappointment or regret or may ruminate over mistakes/things that could not be achieved (despair) [40]. A higher frequency of guilt feelings because of *“Divorce/break up”* and *“Not achieving something/failure”* in old age, of course, is also a consequence of time. If you are older, you simply had more ‘opportunities’ of not achieving something/failure or getting divorced/breaking up (because of statistically more and/or longer relationships) and thus may experience more corresponding feelings of guilt.

According to the findings on male and female participants, participants of the different age groups also showed many similarities regarding reasons for feeling guilty. The supercategory *“Feelings of guilt related to negative self-attributions/flaws”*, for example, was very frequent in participants of all four age groups. It is again important to note that the identified differences are just differences in the frequency; all classified supercategories and the majority of classified categories of stated reasons for feeling guilty were also found in participants of all age groups.

### Limitations

Our study has some limitations. First, we chose to use a web-based survey to collect information on reasons for feelings of guilt, as we expected that this approach would make it easier for people to share such sensitive personal information (compared to approaches such as telephone or face-to-face interviews in online surveys, people are able to anonymously provide written information). Additionally, recruitment was supported by a fieldwork agency with an online panel of registered users to reach out for many potential participants. On the one hand, these data collection and recruitment procedures indeed enabled us to gather information on reasons for feelings of guilt from a significant number of adults. On the other hand, the procedures certainly did not have produced findings representative of the German general adult population, as we, for example, did not reach out for people without access to the internet/to online surveys and/or without corresponding digital competences.

Second, we asked for reasons currently and in the past experienced feelings of guilt in a very simple manner by using the above stated open-ended questions. This ‘free-recall’ approach allowed participants to state all the reasons that came to their mind and allowed us to obtain an unrestricted impression of the wide variety of different reasons for feelings of guilt. It is, however, possible that the freely stated reasons are rather limited to the subjectively most important/severe ones. A ‘cued-recall’ approach in asking for reasons (e.g., *‘Do you currently have feelings of guilt/Have you ever had feelings of guilt because of reason A, reason B, … reason Z?’*(Yes/No)) may have enabled/motivated participants to state more reasons including also less important/severe ones. In addition, participants, of course, were only able to report reasons for feeling guilty they were conscious about. However, as feelings are also driven by unconscious motives, wishes, needs, etc., this study design only allowed to draw conclusions on conscious reasons of feeling guilty. Moreover, without any given information on what guilt is/isn’t (we did not provide any definition, description, vignette etc. – again – to obtain a rather unrestricted impression of feelings of guilt in German adults), it is also possible that some participants may have confused certain things (e.g., feelings of guilt with feelings of shame), and this may have also influenced the participants’ answers on reasons for experienced feelings of guilt.

Third, reasons for feeling guilty stated by people in a certain population/country, of course, are dependent on specific characteristics or circumstances of this population/country. *‘Combat-related guilt’*—a specific type of guilt related to things done/not done/experienced etc. in combat missions –, for example, was not found among the reasons for feeling guilty in our German sample of adults. It is, however, likely that this type of guilt occurs significantly more often in populations/countries, in which more adults serve in the army and in which more adults are involved in (more/more severe) military operations.

Finally—as stated above—, because of the large number of formed (super-)categories of reasons for feeling guilty and the small number of cases in a high number of such categories (especially in certain age- and gender-groups; *see *Table 3), we were not able to perform any convincing inferential statistical analyses on observed group differences in the frequency/percentage of the formed (super-)categories of reasons for feeling guilty.

Due to the limitations, generalizations about the German general adult population should be made with caution.

## Conclusions

Irrespective of these limitations, we think that our findings derived from a large sample of adults are robust enough to suggest that adults can feel guilty for a wide variety of different reasons. Important reasons for guilt feelings may be *“Telling lies/withholding truth/information”* and *“Not spending (enough) time with family (members)/Not taking (enough) care of family (members)/not being there for family (members)”.* Our findings may also empirically support the importance of some specific types of guilt discussed in the literature, such as *‘interpersonal guilt’* or *‘parental guilt’*. Other potentially relevant ‘sources’ for feeling guilty, such as committing sins/misconduct in the eyes of God (guilt feelings explicitly referred to as *‘religious beliefs’*) or a *‘subjectively perceived more general responsibility’* (e.g., for society, for humankind, for the problems of the world), were of minor importance, at least in our study.

Male and female participants as well as participants of different ages showed many similarities but also some differences in stated reasons for feeling guilty. Regarding our results, women, for example, may more often experience feelings of guilt related to family members, children and to some kind of general responsibility for the wellbeing of others, whereas men may feel guilty more often because of some kind of misconduct/mistakes being made or because of difficulties in marriage/relationship. Additionally, important reasons for feeling guilty*, such as “telling lies/withholding truth/information”* and *“misbehavior towards/bad thinking of someone”,* may become less relevant in older age (60+ years). Potential gender- and age-related differences in reasons for feeling guilty, however, should be interpreted only very carefully, avoiding potential blanket stereotypes.

## Data Availability

The raw data of this study cannot be shared for privacy restrictions. Answering to open-ended questions on reasons for feelings guilty, some participants provided comprehensive confidential private information that cannot be made publicly available. However, a minimum dataset not including the raw answers of the participants is available upon reasonable request directed to Prof. Dr. Tobias Luck (tobias.luck@fh-erfurt.de).

## Footnotes

1 Due to the large number of different specific types of guilt, we are unfortunately not able to provide detailed information on each one of them in this article, but will come back on some types later in the discussion section.
